# Disseminated Herpes Zoster Leading to Orbital Apex Syndrome: A Case of MRI-Negative Cranial Neuropathy

**DOI:** 10.7759/cureus.101598

**Published:** 2026-01-15

**Authors:** Brock M Davis, Matthew Waite, Ethan Taylor, Jarod Meecham, Harrison Gregory

**Affiliations:** 1 Dermatology, Northeast Regional Medical Center, Kirksville, USA; 2 Dermatology, A.T. Still University, Kirksville, USA

**Keywords:** disseminated zoster, herpes zoster ophthalmicus, herpes zoster virus, orbital apex syndrome, steroid induced immunosuppression, vzv mri findings

## Abstract

Varicella-zoster virus (VZV) establishes latency in neuronal ganglia following primary infection and can later reactivate as herpes zoster. Disseminated zoster may occur in some cases and is characterized by vesicular lesions appearing beyond the initially affected dermatome. When the ophthalmic branch of the trigeminal nerve is involved, herpes zoster ophthalmicus can develop and may lead to a range of ocular complications. Neurological sequelae are less common, and orbital apex syndrome represents a rare but potentially severe manifestation. This syndrome involves dysfunction of the optic nerve and adjacent cranial nerves within the optic canal and superior orbital fissure, with possible vision loss and ophthalmoplegia. We describe a case of disseminated VZV infection complicated by orbital apex syndrome with cranial nerve III palsy in an immunosuppressed patient, illustrating the diagnostic challenges encountered when clinical findings evolve before confirmatory imaging.

## Introduction

Varicella-zoster virus (VZV), the causative agent of chickenpox (varicella), establishes latency in neuronal ganglia following primary infection, which typically presents as a generalized pruritic rash. Reactivation manifests as herpes zoster (shingles), a painful dermatomal rash [1]. While shingles most commonly remains localized, disseminated zoster, commonly defined as ≥10-12 vesicles outside the primary dermatome within seven to 14 days of onset, or more broadly as multi-dermatomal or widespread vesicular involvement, complicates approximately 2% of cases [2]. Herpes zoster ophthalmicus (HZO) arises when the virus involves the ophthalmic branch of the trigeminal nerve, resulting in vesicular lesions affecting the forehead, scalp, and potentially the eye itself [3]. Ocular complications may include vision loss, loss of corneal sensation, ophthalmoplegia, and uveitis [4,5].

Neurologic sequelae of HZO are less common but may be severe. Orbital apex syndrome (OAS) is characterized by dysfunction of the optic nerve and one or more cranial nerves (III, IV, V1, and VI) within the optic canal and superior orbital fissure, often resulting in vision loss and ophthalmoplegia. Involvement of cranial nerve III is particularly uncommon in HZO-associated OAS, occurring in fewer than 10% of reported cases [6]. Although OAS is frequently associated with inflammatory changes on magnetic resonance imaging (MRI), early or imaging-negative presentations have been described and may complicate timely diagnosis.

We report a case of disseminated VZV infection complicated by OAS with cranial nerve III palsy in an immunosuppressed patient, highlighting an atypical clinical course and the diagnostic challenge posed by MRI-negative cranial neuropathy.

## Case presentation

**Figure 1:**
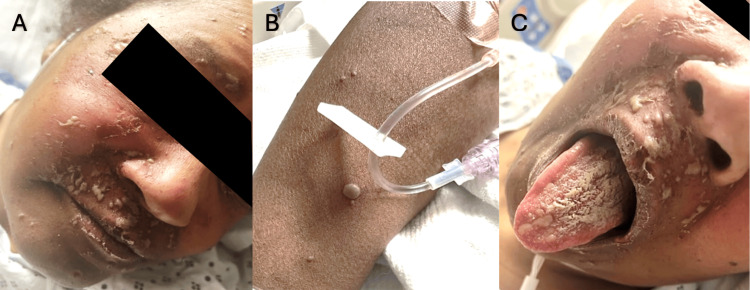
Cutaneous and mucosal manifestations of varicella-zoster virus (VZV) (A) The characteristic vesicular rash distributed along the V1 and V2 dermatomes, consistent with herpes zoster ophthalmicus and maxillary involvement. (B) Discrete vesicles on the right upper extremity. (C) displays oral mucosal involvement with the formation of vesicles and ulcerations on the dorsal surface of the tongue. The presence of vesicles in multiple dermatomes and mucosal surfaces indicates disseminated VZV infection.

A 57-year-old female with Sjögren’s syndrome, hypothyroidism, and systemic lupus erythematosus presented with a six-day history of a progressively spreading vesicular rash. Lesions initially appeared on the right upper extremity and subsequently involved the trunk, genitalia, face, and oral mucosa (Figure 1). Mucosal involvement developed after the onset and progression of cutaneous lesions. She was receiving chronic immunosuppressive therapy with hydroxychloroquine, mycophenolate, methotrexate, and prednisone.

Upon admission, the patient was febrile and tachycardic. Initial laboratory evaluation demonstrated markedly elevated lactic acid and procalcitonin levels (Table 1), raising concern for a systemic infectious process, including sepsis, particularly in the setting of immunosuppression. Broad-spectrum antimicrobial therapy was initiated empirically.

**Table 1:** Laboratory values upon admission with reference ranges

Laboratory Parameter	Value Upon Admission	Reference Range
Lactic Acid	10.5 mmol/L	0.5-2.0 mmol/L
Procalcitonin	4.7 ng/mL	<0.1 ng/mL

On hospital day two, the patient developed acute neurologic changes, including altered mental status with auditory and visual hallucinations, as well as a complete right cranial nerve III palsy. Neurologic examination revealed ptosis, impaired extraocular movements, and a fixed, dilated, nonreactive right pupil. These findings raised concern for central nervous system involvement, including infectious or inflammatory etiologies.

Computed tomography (CT) of the head, CT angiography of the head and neck, MRI of the brain and orbits, and magnetic resonance venography were unremarkable. Lumbar puncture demonstrated cloudy cerebrospinal fluid (CSF), and polymerase chain reaction testing was positive for VZV. Quantitative CSF parameters were not available in the medical record.

The patient was treated with intravenous acyclovir at 10 mg/kg every eight hours, dexamethasone, and meropenem. Following initiation of therapy, her mental status returned to baseline, and extraocular movements improved. She was discharged on a 10-day course of oral acyclovir with a prednisone taper and scheduled follow-up with rheumatology, ophthalmology, and neurology.

## Discussion

Disseminated herpes zoster typically presents with vesicular lesions across multiple dermatomes following an initial localized rash. This case is notable for two features: the atypical sequence of cutaneous involvement, in which peripheral lesions preceded facial involvement, and the development of OAS in the absence of corresponding MRI abnormalities. Together, these findings highlight the diagnostic complexity of herpes zoster in immunosuppressed patients.

**Figure 2:**
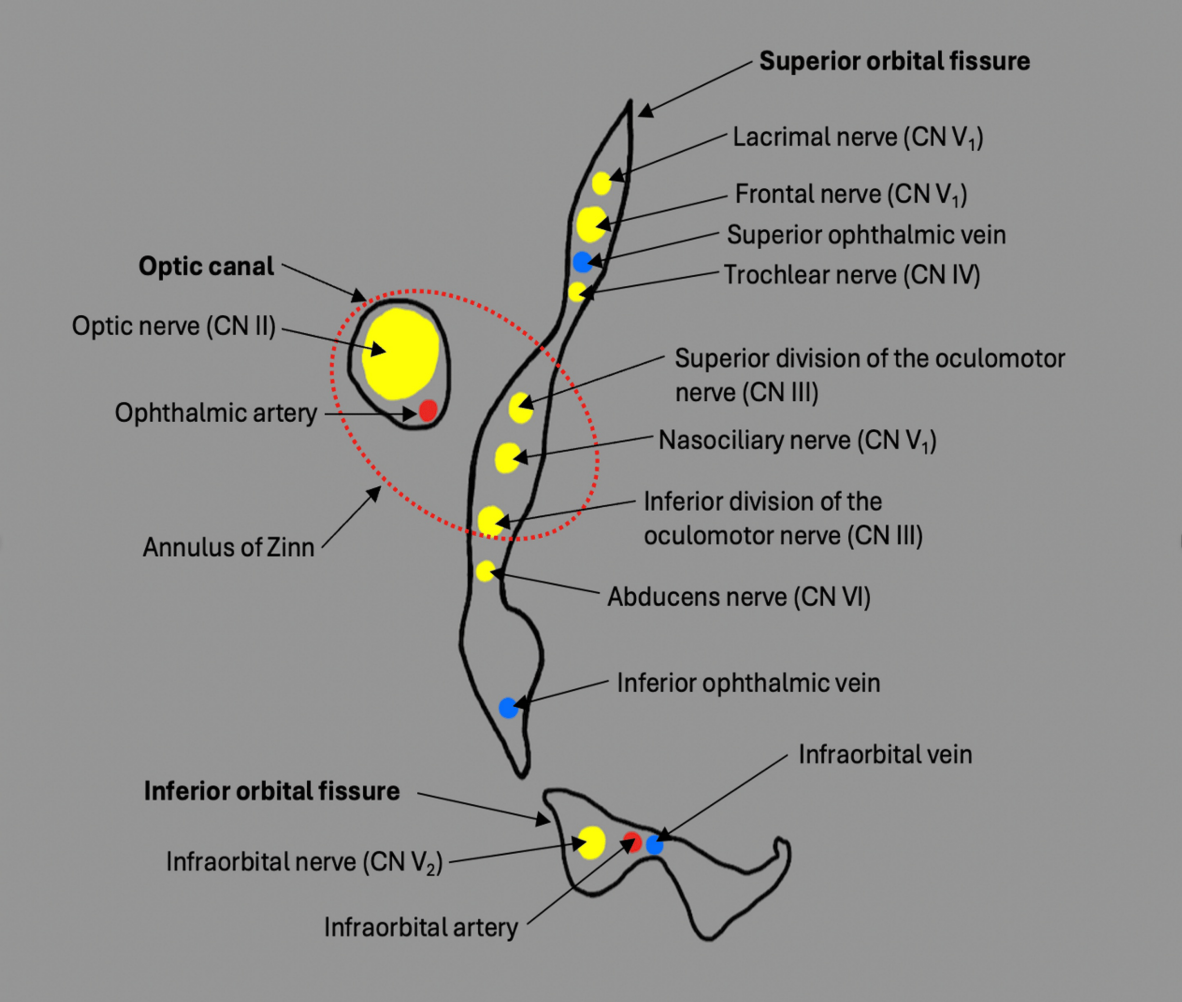
Anatomical structures of the orbit and hypothesized pathways of viral spread This schematic illustrates the complex relationships between the optic nerve, cranial nerves, and adjacent vascular structures within the orbital apex. The proposed routes of varicella-zoster virus dissemination are speculative and are presented as conceptual hypotheses rather than established mechanisms. This figure was created by the authors and does not require attribution or permission.

Immunosuppressed individuals are at increased risk for disseminated VZV infection and may exhibit atypical clinical presentations compared with immunocompetent hosts [7]. The pattern of lesion progression observed in this case differs from the more typical centripetal spread from a primary dermatome. While similar atypical or non-dermatomal presentations have been reported in immunocompromised patients, the mechanism underlying this pattern remains unclear. Accordingly, this observation should be interpreted cautiously and is best regarded as hypothesis-generating rather than indicative of a defined pathophysiologic process [8]. As illustrated in Figure 2, the complex anatomic relationships of cranial nerves and vascular structures within the orbit may represent potential routes for viral spread; however, these proposed pathways are speculative.

Orbital apex involvement in herpes zoster is rare and is most often associated with inflammatory changes on MRI [9]. In this case, however, neuroimaging of the brain and orbits was unremarkable despite clear clinical evidence of cranial nerve III palsy and encephalopathy. Although uncommon, MRI-negative cases of zoster-associated OAS have been described in the literature and may reflect early disease or direct viral involvement without overt radiographic inflammation [10]. This underscores the importance of maintaining clinical suspicion even when imaging findings are unrevealing.

Given the absence of radiographic abnormalities, CSF analysis played a critical role in establishing the diagnosis. Polymerase chain reaction testing confirmed VZV infection, supporting the diagnosis of VZV-associated encephalitis with orbital apex involvement [10]. This case highlights the diagnostic value of early lumbar puncture in patients with unexplained neurologic findings, particularly when imaging is inconclusive.

Prompt initiation of high-dose intravenous acyclovir is essential in the management of neurologic complications of VZV. Although the use of adjunctive corticosteroids remains controversial, they may be considered in select cases involving severe neurologic manifestations, including OAS, to mitigate inflammation [11]. In this patient, early antiviral therapy with adjunctive corticosteroids was associated with rapid neurologic improvement; however, conclusions regarding therapeutic efficacy should be interpreted cautiously, given the limitations inherent to a single case.

## Conclusions

HZO complicated by OAS is an exceptionally rare and potentially devastating condition, particularly in immunocompromised patients. This case highlights the importance of early recognition and intervention, as delays may lead to severe sequelae such as permanent vision loss or neurological deficits. The atypical presentation, marked by a reverse pattern of skin lesion progression and the absence of MRI abnormalities despite clear clinical evidence of OAS, underscores the diagnostic challenges posed by VZV reactivation in immunosuppressed individuals. Although this report represents a single case and conclusions regarding pathophysiology and management should be interpreted cautiously, it emphasizes the necessity of maintaining a low threshold for CSF analysis when neurologic involvement is suspected, particularly when imaging is inconclusive. Prompt initiation of antiviral therapy, with adjunctive corticosteroids considered in select cases, can be associated with substantial neurologic recovery, as demonstrated by this patient’s return to baseline mental status and resolution of ophthalmoplegia. A multidisciplinary approach involving ophthalmology, neurology, and rheumatology remains essential for comprehensive management and follow-up.
